# The Haiti Breast Cancer Initiative: Initial Findings and Analysis of Barriers-to-Care Delaying Patient Presentation

**DOI:** 10.1155/2013/206367

**Published:** 2013-06-05

**Authors:** Ketan Sharma, Ainhoa Costas, Ruth Damuse, Jean Hamiltong-Pierre, Jordan Pyda, Cecilia T. Ong, Lawrence N. Shulman, John G. Meara

**Affiliations:** ^1^Program in Global Surgery and Social Change, Harvard Medical School, 25 Shattuck Street, Boston, MA 02115, USA; ^2^Department of Plastic and Oral Surgery, Children's Hospital Boston, 300 Longwood Avenue, Enders 1, Boston, MA 02115, USA; ^3^Duke University School of Medicine, 201 Trent Drive, Durham, NC 27715, USA; ^4^Clinique Bon Sauveur, Zanmi Lasante, Cange, Haiti; ^5^University of Tennessee Health Science Center, 910 Madison Avenue, Memphis, TN 38163, USA; ^6^Dana Farber Cancer Institute, Brigham and Women's Hospital, 450 Brookline Avenue, Boston, MA 02115, USA

## Abstract

*Background.* In Haiti, breast cancer patients present at such advanced stages that even modern therapies offer modest survival benefit. Identifying the personal, sociocultural, and economic barriers-to-care delaying patient presentation is crucial to controlling disease. *Methods.* Patients presenting to the Hôpital Bon Sauveur in Cange were prospectively accrued. Delay was defined as 12 weeks or longer from initial sign/symptom discovery to presentation, as durations greater than this cutoff correlate with reduced survival. A matched case-control analysis with multivariate logistic regression was used to identify factors predicting delay. *Results.* Of *N* = 123 patients accrued, 90 (73%) reported symptom-presentation duration and formed the basis of this study: 52 patients presented within 12 weeks of symptoms, while 38 patients waited longer than 12 weeks. On logistic regression, lower education status (OR = 5.6, *P* = 0.03), failure to initially recognize mass as important (OR = 13.0, *P* < 0.01), and fear of treatment cost (OR = 8.3, *P* = 0.03) were shown to independently predict delayed patient presentation. *Conclusion.* To reduce stage at presentation, future interventions must educate patients on the recognition of initial breast cancer signs and symptoms and address cost concerns by providing care free of charge and/or advertising that existing care is already free.

## 1. Introduction

Breast cancer remains the most common cancer and most common cause of cancer-related death amongt women worldwide [1]. While incidence rates have historically been higher in the developed world, recent evidence suggests an alarming increase in both incidence and mortality in low-income developing countries (LIDCs) [2]. Coupled with stable-to-declining age-standardized mortality rates in developed countries [3], an increasing number of preventable breast cancer deaths will continue to shift to LIDCs worldwide. 

Within LIDCs, the problem is further exacerbated as relative survival rates (approximated as the complement of the mortality-to-incidence ratio [4]) are lower due to both advanced stage at presentation and inaccessibility of potentially curative therapy [2, 5]. For example, in one study of sub-Saharan Africa, 90% of breast cancer patients presented with stage III or IV disease, a median tumor size of 10 cm, and palpable nodal metastasis [6]. These patterns of disease can be so advanced that even optimal western therapy may offer minimal survival benefit. This is distinctly different than in the developed world, where the majority of women present with early-stage disease, and more than 80% survive at least for 5 years [7]. Furthermore, these advanced stages are chiefly the result of delayed presentation [8]. Delays to seek for care longer than 12 weeks have been shown in a systematic review involving 87 studies to correlate with decreased 5-year survival, though it is likely that delay is a continuous variable in regard to outcome [9]. Hence, to maximize benefit, future interventions to control breast cancer within LIDCs must address delayed presentation in addition to extending access to modernized care.

Delayed patient presentation refers to a prolonged interval between discovery of initial sign/symptom and presentation to a qualified medical provider. A recent systematic review by our group examining causes of patient delay within LIDCs grouped these variables into three potentially overlapping categories: personal, sociocultural, and economic [10]. Personal factors are patient characteristics not amenable to intervention (i.e., age or medical history), while sociocultural variables include breast cancer awareness and stigma of disease, and economic variables include occupation and cost of care. To effectively prevent delayed patient presentation in LIDCs, the specific causative variables contributing to delay must be identified out of the myriad of potential contributory ones (Table 1).

One LIDC with a paucity of research is Haiti, which may be “faced with the worst health and human development statistics in the region” [11]. Currently, Haiti is estimated to exhibit a breast cancer incidence of 4.4 per 100,000 females per year and mortality of 2.0 per 100,000 females per year for an approximate relative mortality of 45% [12]. In comparison, the United States has a higher incidence of 121.2 per 100,000 females per year and a higher mortality of 23.5 per 100,000 females per year, but a much lower approximate relative mortality of 19% [13]. Unfortunately, Haiti's current epidemiological estimates are modeled from data of similar and surrounding nations. To address the concerning lack of in-country data, the Program in Global Surgery and Social Change (PGSSC) at Harvard Medical School, in conjunction with Partners In Health and Zanmi Lasante (PIH/ZL), launched the Haiti Breast Cancer Initiative (HBCI): the first prospective hospital-based Haitian breast cancer registry.

Therefore, the purpose of this study is to use initial HBCI results to (1) identify the personal, sociocultural, and economic barriers-to-care that delay breast cancer presentation within this Haitian patient population and (2) recommend personalized interventions that preempt patient delay, reduce stage at presentation, and consequently improve mortality, in an effort to control the burden of breast cancer disease within Haiti.

## 2. Materials and Methods

### 2.1. Study Population and Design

The HBCI is a prospective, hospital-based breast cancer registry at the Breast Clinic of the Partners In Health (PIH)/Zanmi Lasante (ZL) Hôpital Bon Sauveur in Cange, Haiti. Any female patient over 18 years old who presents for care is offered the chance to be enrolled; informed consent is obtained from any willing participant. The weekly Breast Clinic, run by one of the senior authors (Ruth Damuse), offers screening, diagnosis, and social work services. Patients are provided with medical treatments such as chemotherapy and may be referred for surgical intervention ranging from biopsy to modified radical mastectomy. All care is provided free of charge.

The database collects standardized data on patient demographics, barriers-to-care, medical history, clinical presentation, diagnostic workup including imaging and pathology, surgical and medical treatments, and outcomes including complications and disease status at points of followup. Regarding barriers-to-care and presentation delay, patients were asked “did any of the following prevent you from coming to clinic sooner?” and provided a list of 14 yes/no answers (Table 3). IRB approval was obtained by both Children's Hospital Boston and PIH/ZL. 

### 2.2. Statistical Analysis

Delayed presentation was defined as 12 weeks or greater from discovery of first breast cancer sign or symptom to initial presentation to a Haitian healthcare provider, in agreement with the existing literature [14] and based on systematically reviewed evidence that durations longer than this correlate with decreased survival, though it is likely that delay is a continuous variable in regard to outcome [9]. Summary statistics were tabulated using *χ* ^2^ to compare proportions and Wilcoxon rank-sum to compare sample medians. A multivariate logistic regression model using patient demographics and survey responses to predict delayed presentation was employed. To be included, potential covariates had to exhibit statistical significance (*P* < 0.05) on univariate comparison between patients with and without delay. The model's accuracy was measured by the Hosmer-Lemeshow Goodness-of-Fit test to test calibration and *c*-statistic to test discrimination. Two-sided *α* = 0.05 indicated significance in all tests. All statistical analyses were performed via commercially-available STATA v11.1 (College Station, TX, USA).

## 3. Results

**Figure 1:**
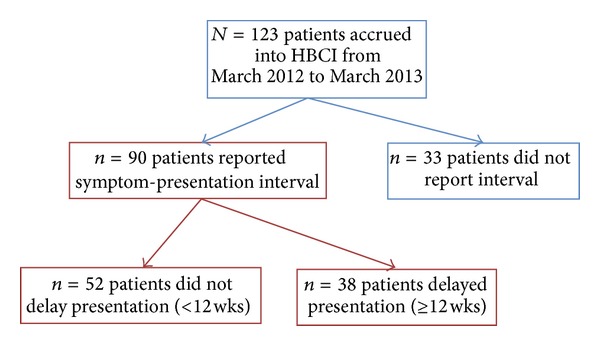
HBCI patient flowchart. Note: patients in red boxes reported symptom-presentation interval and formed the basis of our study.

From March 1, 2012 to March 1, 2013, *N* = 123 breast cancer patients who presented to the Breast Clinic in Cange agreed to enroll in the HBCI. Of this total, 90 patients reported a symptom-presentation interval and therefore formed the basis for this study. Of these, *n* = 52 (58%) presented to a healthcare provider less than 12 weeks after discovering their initial breast-cancer-related sign/symptom (median duration: 1 week, IQR: 1–4 weeks), whereas *n* = 38 (42%) waited longer than 12 weeks and therefore delayed their patient presentation (median duration: 26 weeks, IQR: 17–77 weeks) (Figure 1).

Several important demographic differences were noted between the two groups. Patients with delay were less likely to have completed secondary school or higher (16% versus 43%, *P* = 0.02). In addition, patients with delay were slightly older (median 49 versus 45 years, *P* = 0.04). There were no statistically significant differences between the two groups with respect to marital status, employment status, breast cancer history, time to reach clinic, or method of reaching clinic (Table 2).

Amongt patients with delay, the three most commonly cited barriers-to-care were: “I wasn't bothered by the mass at first” (18/38, 47%), “I did not know I needed to see a doctor and thought it would go away” (13/38, 34%), and “I thought treatment might be too expensive” (8/38, 21%). Furthermore, when comparing survey responses between the two groups, patients with delay were significantly more likely to claim the following as barriers-to-care: “I was afraid of being examined by a doctor or other health official” (8% versus 0%, *P* = 0.04), “I was not bothered by the mass at first” (47% versus 10%, *P* < 0.01), “I was afraid of possibly dying if my breast was removed” (11% versus 0%, *P* = 0.02), and “I thought treatment might be too expensive” (21% versus 6%, *P* = 0.03) (Table 3).

**Figure 2:**
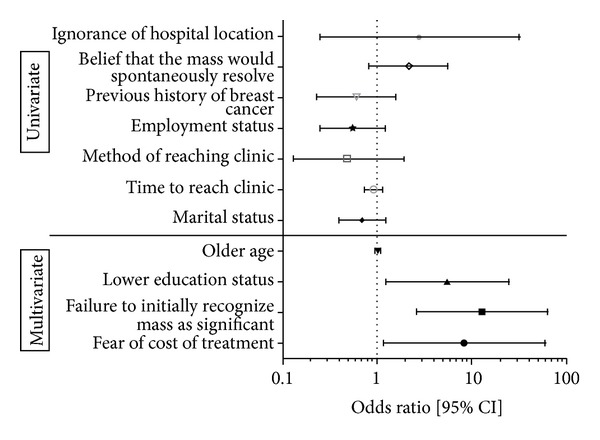
Logistic regression analysis. Note: univariate logistic regression for covariates not meeting the inclusion criteria into the multivariate model is shown for reference. Only covariates that exhibit significance in the multivariate analysis are considered as independent risk factors for delayed patient presentation (barriers-to-care). Multivariate logistic regression model: *c* statistic = 0.807, concordant pair percentage = 80.7%, discordant pair percentage = 19.3%, Hosmer-Lemeshow Goodness-of-Fit test: *χ* ^2^ = 3.95, *P* = 0.86 (df = 8).

Finally, a multivariate logistic regression model was constructed using the following covariates as they exhibited statistical significance on univariate comparison: age, lower education status (defined as receiving no education or attending primary school only), fear of examination (“I was afraid of being examined by a doctor or other health official”), failure to initially recognize mass as important (“I was not bothered by the mass at first”), fear of treatment (“I was afraid of possibly dying if my breast was removed”), and fear of cost of treatment (“I thought treatment might be too expensive”). Of these, three variables were shown to independently increase the odds of delaying presentation: lower education status (OR = 5.6, *P* = 0.03), failure to initially recognize mass as important (OR = 13.0, *P* < 0.01), and fear of cost of treatment (OR = 8.3, *P* = 0.03). Moreover, the model showed an excellent ability to predict patient delay (*c* statistic = 0.807, concordant percentage = 80.7%) and fit the observed data well (Hosmer-Lemeshow Goodness-of-Fit test: *χ* ^2^ = 3.95, *P* = 0.86) (Figure 2).

## 4. Discussion

Delayed patient presentation refers to a prolonged interval (≥12 weeks) between discovery of initial breast-cancer-related sign/symptom and presentation to the first qualified medical provider available. Patient delay has been shown to be a major contributor toward advanced stage at presentation and relatively increased mortality in both developing countries and underserved populations in developed nations [2, 9, 15]. To our knowledge, this study is the first evaluation of potential personal, sociocultural, and economic risk factors for delayed patient presentation (or barriers-to-care) within Haiti. 

Our findings suggest that patient delay is a prevalent, complex, and multifactorial phenomenon that spans sociocultural and economic domains. Notably, we observed a profound difference in symptom-presentation duration between patients without delay (median: 1 week, IQR 1–4 weeks) and patients with delay (median: 26 weeks, IQR: 17–77 weeks). This bimodal distribution suggests the existence of two separate groups within our patient population. When comparing these groups on univariate analysis, the following were significantly associated with delay: lower education status, fear of examination, failure to initially recognize mass as important, fear of treatment, fear of cost of treatment, and (to a lesser extent) older age (Table 2). 

Of these potential barriers-to-care, only three survived the most rigorous multivariate logistic regression analysis. Specifically, lower education status (defined as receiving no formal education or attending primary school only) increased the odds of delay almost sixfold, while fear of cost of treatment increased the odds more than eightfold, and failure to initially recognize mass as important increased the odds thirteenfold. Hence, out of the myriad of potential contributory factors, these three constitute the most meaningful barriers-to-care in this setting (Figure 2).

Similar research conducted in other LIDCs has yielded varying results. One study in South India found that being unmarried and lower education level were associated with delayed presentation [16]. Another study of Iranian women observed an association with positive family history of breast cancer [17]. A case series in Ghana listed privious medical consultation, ignorance about breast cancer, and fear of mastectomy as the three most cited factors leading to delay [18]. These heterogeneous results may stem from variations in study methodology and/or differences between LIDCs themselves, where cultures and economies can vary tremendously. A recent systematic review by our group observed strong evidence for personal and economic factors related to patient delay and moderate evidence for sociocultural ones in LIDCs, but noted that, while the current evidence for such sociocultural factors remains moderate, this may reflect the current paucity of (high-quality) studies, and future research is required to ascertain the true relationship between sociocultural variables and patient delay in the developing world [10].

Our study suffers from several limitations. First, the HBCI is a hospital-based registry that recruits patients who present for treatment in the PIH/ZL system. As such, the likelihood of missing patients who present to other facilities or do not present at all remains high. These missed patients may have different rationales for not presenting for care, producing potential selection bias. For instance, it is conceptually feasible that HBCI patients are less dissuaded by costs of travel and treatment than patients who did not present at all. Thus, our study may understate the importance of economic barriers-to-care. However, a comprehensive population-based registry is currently not feasible given Haiti's profound resource constraints. Second, patient delay was assessed retrospectively at the point of presentation, introducing possible measurement bias. Patients may have misestimated the duration of delay, or may potentially have presented to other sites first. However, this bias is less likely to affect our results, given the extreme difference in symptom-presentation duration between the two patient groups (median 1 week versus 26 weeks, *P* < 0.01) and the large catchment area and free nature of care at the Hôpital Bon Sauveur [19]. Furthermore, a prospective assessment of delay would be unethical and infeasible, so this bias is inherently unavoidable. Third, although the possibility of unknown confounding remains, this study utilized high-powered multivariate logistic regression controlling for demographics and other potential barriers-to-care, so this potential is minimized as best possible.

The findings of this study can be used to optimize future interventions aimed at preempting patient delay in Haiti. First, from an advocacy standpoint, the Haitian Ministry of Health and PIH/ZL could embark on an educational and public relations campaign that educates women on how to better recognize the initial signs and symptoms of breast cancer and emphasizes the need for presentation to a healthcare provider for any suspicious findings. It is important to emphasize to women that a mass or lump that is not initially bothersome may nonetheless portend cancer and thereby require proper evaluation. In particular, in the senior authors' (Ruth Damuse and Ainhoa Costas) experience, lack of pain tends to be the most common reason for patients not to present for evaluation, though future surveys are required to confirm this. Second, the real and perceived economic barriers to breast cancer care must be addressed. Both the actual and perceived costs of treatment must be reduced, either by reducing the cost itself or by advertising that treatment at certain sites is already free (PIH/ZL offers free treatment at Cange). Potential interventions here could include mass media campaigns to educate Haitians on breast cancer and train community health workers to teach breast self-exam (BSE). Though BSE has not been shown to improve survival as an asymptomatic screening tool [20], current screening alternatives in Haiti are extremely limited, and BSE education may teach this patient population to better recognize the initial signs and symptoms of breast cancer and present earlier in the natural history of their disease; this issue requires further prospective study. PIH/ZL is currently training its community health workers to teach cancer understanding and broadcast information about breast cancer prevention and treatment via local radio stations. Future research must analyze the effectiveness of these interventions on enhancing breast cancer awareness and include rigorous cost-effectiveness analysis to account for resource constraints.

## 5. Conclusion

To our knowledge, this constitutes the first prospective breast cancer study in Haiti. Since patients in LIDCs such as Haiti present with such advanced disease, controlling the burden of breast cancer globally will require not solely extending access to modern therapies, but also developing interventions that help patients present sooner in the natural course of their disease. To help achieve this goal, our research identifies targets for the most pertinent personal, sociocultural, and economic barriers-to-care. Only by educating women on the signs/symptoms of breast cancer and the importance of prompt treatment and by decreasing the perceived and actual cost of treatment will it be possible to reverse the alarming increase and human toll of breast cancer in Haiti.

## Figures and Tables

**Table 1:** Potential risk factors for delayed patient presentation (barriers-to-care).

Personal	Sociocultural	Economic
Age Ethnicity Marital status Clinical presentation Personal history Family history	Breast cancer awareness **Failure to initially recognize mass as important** Lack of breast-self exam (BSE) use Alternative therapy use Fear of examination Fear of treatment Stigma of disease Denial/anxiety	Fear of cost of travel **Fear of cost of treatment** Obligations at home/work Access to health systems Income status **Education status**

Note: variables in **bold** were shown to predict patient delay in the multivariate logistic regression analysis (see Figure 2).

**Table 2:** Patient characteristics.

	Presentation not delayed *N* = 52 (58%)	Presentation delayed *N* = 38 (42%)	*P*-value
**Age (yrs)** ^ 1^	**45 (39–53)**	**49 (43–62)**	**0**.**04**
**Symptom-presentation duration(wks)** ^ 1^	**1 (1–4)**	**26 (17–77)**	**<0**.**01**
Marital status			
Single, divorced, widowed	18 (35%)	17 (45%)	0.48
Married	21 (40%)	15 (39%)	
Not reported	13 (25%)	6 (16%)	
**Education status**			
**None**	**5 (10%)**	**11 (29%)**	
**Primary school**	**19 (37%)**	**13 (34%)**	**0**.**02**
**Secondary school**	**16 (31%)**	**6 (16%)**	
**University**	**6 (12%)**	**0 (0%)**	
**Not reported**	**6 (12%)**	**8 (21%)**	
Employment status			
Unemployed	30 (58%)	28 (74%)	0.29
Employed	20 (38%)	9 (24%)	
Not reported	2 (4%)	1 (3%)	
Prior history of breast cancer			
None	47 (90%)	36 (95%)	0.22
Yes	0	1 (3%)	
Not reported	5 (10%)	1 (3%)	
Time to reach clinic (hrs)^1^	3 (3-4)	3 (3-4)	0.71
Method of reaching clinic			
Walk	0 (0%)	1 (3%)	
Car	3 (7%)	3 (8%)	0.53
Public transportation	40 (89%)	32 (86%)	
Not reported	2 (4%)	1 (3%)	

^1^Median (interquartile range).

**Table 3:** Barriers-to-care survey: *did any of the following prevent you from coming to clinic sooner*?

Survey response	Patients without delay (*N* = 52)	Patients with delay (*N* = 38)	*P*-value
**I was afraid of being examined by a doctor or other health official**	**0 (0%)**	**3 (8%)**	**0.04**
I had or knew someone who had a bad experience at a hospital before	0 (0%)	0 (0%)	n/a
**I was not bothered by the mass at first**	**5 (10%)**	**18 (47%)**	**<0.01**
I did not know I needed to see a doctor and thought it would go away	10 (20%)	13 (34%)	0.11
I did not know where an appropriate medical facility was	1 (2%)	2 (5%)	0.38
I was too busy at my home or job	0 (0%)	1 (3%)	0.24
I was afraid of treatments, including potentially losing my breast	0 (0%)	2 (5%)	0.10
I did not want anyone knowing that I had a mass	0 (0%)	1 (3%)	0.24
I tried another treatment or visited another healer first	0 (0%)	1 (3%)	0.24
**I was afraid of possibly dying if my breast was removed**	**0 (0%)**	**4 (11%)**	**0.02**
I was afraid it might be cancer	0 (0%)	2 (5%)	0.10
**I thought treatment might be too expensive**	**3 (6%)**	**8 (21%)**	**0.03**
It was too expensive to travel to the clinic	0 (0%)	0 (0%)	n/a
The clinic was too far away for me to travel to	0 (0%)	0 (0%)	n/a
